# Nesfatin-1: A Novel Diagnostic and Prognostic Biomarker in Digestive Diseases

**DOI:** 10.3390/biomedicines12081913

**Published:** 2024-08-20

**Authors:** Adriana-Cezara Damian-Buda, Daniela Maria Matei, Lidia Ciobanu, Dana-Zamfira Damian-Buda, Raluca Maria Pop, Anca Dana Buzoianu, Ioana Corina Bocsan

**Affiliations:** 1 Pharmacology, Toxicology and Clinical Pharmacology Laboratory, “Iuliu Hațieganu” University of Medicine and Pharmacy, 400012 Cluj-Napoca, Romania; adriana.ceza.damianbuda@elearn.umfcluj.ro; 2 Department of Internal Medicine, “Iuliu Hațieganu” University of Medicine and Pharmacy, 400012 Cluj-Napoca, Romania; dmatei68@gmail.com (D.M.M.); ciobanulidia@yahoo.com (L.C.); 3 Regional Institute of Gastroenterology and Hepatology, 400162 Cluj-Napoca, Romania; danadamiancluj@yahoo.com; 4 Pharmacology, Toxicology and Clinical Pharmacology, Department of Morphofunctional Sciences, “Iuliu Haţieganu” University of Medicine and Pharmacy, Victor Babeș, No 8, 400012 Cluj-Napoca, Romania; abuzoianu@umfcluj.ro (A.D.B.); bocsan.corina@umfcluj.ro (I.C.B.)

**Keywords:** nesfatin-1, anti-inflammatory, antioxidant, anorexigenic, digestive disorders, inflammatory disorders

## Abstract

Nesfatin-1, deriving from a precursor protein, NUCB2, is a newly discovered molecule with anti-apoptotic, anti-inflammatory, antioxidant, and anorexigenic effects. It was initially identified in the central nervous system (CNS) and received increasing interest due to its energy-regulating properties. However, research showed that nesfatin-1 is also expressed in peripheral tissues, including the digestive system. The aim of this review is to give a résumé of the present state of knowledge regarding its structure, immunolocalization, and potential implications in diseases with inflammatory components. The main objective was to focus on its clinical importance as a diagnostic biomarker and potential therapeutic molecule in a variety of disorders, among which digestive disorders were of particular interest. Previous studies have shown that nesfatin-1 regulates the balance between pro- and antioxidant agents, which makes nesfatin-1 a promising therapeutic agent. Further in-depth research regarding the underlying mechanisms of action is needed for a better understanding of its effects.

## 1. Introduction

Identified in 2006 by a research group led by Oh-I [1] in the hypothalamic nuclei, nesfatin-1 was considered from the very beginning a potent anorexigenic neuropeptide. 

Nesfatin-1 is composed of 82 amino acids and derives from nucleobindin-2, NUCB2, its precursor protein, consisting of 420 amino acids (396 amino acids and a 24 amino acid long signal peptide). To become activated, it undertakes posttranslational processing, which leads to the formation of three smaller biologically active fragments: nesfatin-1 (residues 1–82), nesfatin-2 (85–163), and nesfatin-3, respectively (166–396) [1]. The nesfatin-1 molecular structure is made of three different domains: the N-terminal part, the middle (responsible for its physiological effects), and the C-terminal part. While nesfatin-1 is directly involved in a wide variety of physiological and pathological processes, the other two peptides have thus far unknown functions [1].

Initially described in the hypothalamic nuclei, which regulate food intake (the supraoptic nucleus, paraventricular nucleus, lateral hypothalamic area, the arcuate nucleus, dorsomedial nucleus, zona incerta), nesfatin-1 immunoreactive cells were soon detected in other areas of the central nervous system (raphe pallidus, raphe obscurus, the accessorius nucleus of the oculomotor, nucleus dorsalis of the vagus nerve) [2,3]. Nesfatin-1 is directly released from the synaptic endings of the vagus nerve, and this might somewhat explain how the central nervous system (CNS) regulates both the secretory and the motor activity of the gastrointestinal (GI) tract [4,5]. Other regions of the CNS characterized by nesfatin-1 expression are the insular cortex, central amygdaloid nucleus, Purkinje cells of the cerebellum, the lumbar and sacral spinal cord, pterygopalatine parasympathetic preganglionic neurons, and some other areas of the brain stem such as locus coeruleus, dorsal vagal complex, ventrolateral medulla, and the Edinger–Westphal nucleus [5].

Since the discovery that nesfatin-1 can penetrate the blood–brain barrier in a non-saturable manner [6], new binding sites in peripheral organs have been detected. Indeed, in addition to its wide CNS distribution, nesfatin-1 has proved to be expressed in a wide variety of peripheral tissues, among which the digestive system is of particular interest [7,8]. The stomach, duodenum, and pancreas showed high nesfatin-1 expression. The main source of NUCB2/nesfatin-1 in the mammalian gut is a subset of endocrine cells located in the lower third and middle portions of the glands of the oxyntic gastric mucosa [9]. Interestingly, Stengel et al. demonstrated that in most of these endocrine cells (X/A cells), nesfatin-1 is co-expressed with the orexigenic hormone, ghrelin. However, the two peptides are stored in two different populations of intracytoplasmic vesicles [10]. 

The results of the studies that focused on the presence of nesfatin-1 in the small intestine are divergent. It was found that higher expression was found in the Brunner’s glands of the duodenum [9]. The glands produce a secretion containing bicarbonate and mucus, thus protecting the duodenum mucosa from acidic chyme while regulating intestinal enzyme activation. Therefore, the presence of nesfatin-1 might suggest a potential role in nutrient absorption and preservation of intestinal wall integrity. It is important to take into consideration the fact that recently nesfatin-1 has been present in both myenteric and submucosal plexuses of the duodenum in normal as well as in gastrectomized rats. This reinforces the assumption that nesfatin-1 could elicit a variety of effects as part of the brain axis. Moreover, in the rat small intestine, nesfatin-1 is mainly expressed in the Paneth cells [11].

On the other hand, nesfatin-1 expression was discovered in the enteroendocrine cells of neonatal rats [12]. As far as the localization in the large intestine is concerned, initial studies using quantitative reverse transcription polymerase chain reaction (RT-qPCR) and Western blot indicated low levels of NUCB2/nesfatin-1 in different species of dogs [13], rats, and mice [9] with distribution varying based on age and segment. However, the results of immunohistochemical studies are rather inconsistent and show species-specific characteristics. In canines, only a very small population of nesfatin-1-immunoreactive cells were identified or had no reactive cells at all [14]. Similarly, in mice and rats, no nesfatin-1 expression was identified [9]. 

Initially, it was revealed that in the rat pancreas, nesfatin-1 is expressed in the beta islet cells. Immunofluorescence studies conducted in humans and rats confirmed that beta cells store nesfatin-1, but the other islet cells (alfa, delta, PP) and the exocrine pancreas do not. Intracellularly, nesfatin-1 is co-stored with insulin, but they have a different subcellular distribution [15].

**Figure 1:**
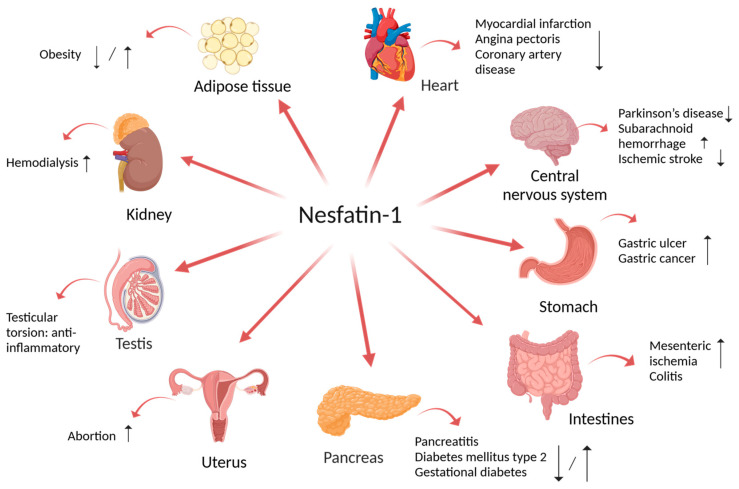
Nesfatin-1: localization and possible role in tissue functions regulation. Created with BioRender.com (accessed on 1 August 2024).

The presence of nesfatin-1 in different amounts in other peripheral tissues such as the cardiomyocytes [16], adipocytes [17], the esophagus [13], liver, kidney, ovaries [18], testis [19], uterus, and epididymis [20] is of great relevance (Figure 1). Its ubiquitous distribution explains the implication of this novel biomarker in the pathogenesis of different diseases.

Despite its wide systemic distribution, the current state of knowledge regarding the mechanisms of action of nesfatin-1 is still limited. There were several attempts to detect specific intracellular or extracellular receptors, but the results were inconclusive. Nonetheless, there is strong evidence that nesfatin-1 may bind to a specific receptor that mediates its functions. By using Iodine 125-nesfatin-1 autoradiography, binding sites for nesfatin-1 were identified both in the brain as well as in peripheral organs such as the stomach, duodenum, jejunum, ileum, and the pancreas, with no signal in the colon [21]. A potential functional cooperation between the biomarker and a G protein-coupled receptor was suggested by the fact that nesfatin-1 increased the Ca^2+^ influx in neurons of the hypothalamus via the activation of the L, P, and Q-Ca^2+^ channels [22].

Currently, there is a series of proposed signaling pathways that mediate the pleiotropic effects of nesfatin-1, as summarized in Table 1.

## 2. Implication of Nesfatin-1 in Multiple Disorders

After the discovery of its expression in a large number of tissues and organs, nesfatin-1 has received increased interest. Extensive research has been conducted to explore its implications in several diseases. Its anti-inflammatory, anti-apoptotic properties can explain the therapeutic potential of nesfatin-1 in a variety of diseases.

The relationship between nesfatin-1 and food intake and metabolic disorders has received special attention. Currently, there are multiple suggested mechanisms for the relationship between nesfatin-1, obesity, and metabolic dysfunction, particularly type 2 diabetes mellitus. Nesfatin-1 seems to regulate vagal neurons and the autonomic functions in the brainstem, midbrain, and hypothalamus. On the other hand, the vagus nerve also modulates nesfatin-1 expression [44]. Vagectomy increased nesfatin-1 levels and abolished the CNS negative control of the nesfatin-1 secretion in mice with induced nonalcoholic fatty liver disease (NAFLD) subjected to Roux-en-Y gastric bypass [45]. The possible implication of CNS activity on body weight regulation is also supported by the finding that the gut–brain axis may play a central role in the regulation of food intake and metabolic processes. Together with ghrelin, nesfatin-1 is now considered one of the main gastrokines involved in the gut–brain axis. Similar to ghrelin, nesfatin-1 gastric expression is regulated by nutritional status, with significantly reduced levels of nesfatin-1 being detected under fasting conditions. The mTOR signaling pathway has been proposed to modulate nesfatin-1 expression and energy balance [46]. Moreover, it has been demonstrated that the anorexigenic function can at least in part be explained by its beneficial effect on lipid metabolism. Nesfatin-1 prevents fat accumulation and accelerates lipid degradation in various tissues [47,48,49]. Even though nesfatin-1 normally inhibits gastric emptying, it has been shown that a high-fat diet stimulates gastric contractility and abolishes the effects of nesfatin-1 [50]. Another factor that influences metabolic homeostasis is food intake. Nesaftin-1 reduces food intake via the negative modulation of dopaminergic neurons, which results in a reduced rewarding feeling related to food ingestion. Taking into consideration that nesfatin-1 exerts its anorexigenic effects even when leptin resistance is present, the neuropeptide can be of therapeutic importance when it comes to the management of obesity [51,52]. Also, its therapeutic potential in eating disorders such as anorexia nervosa should not be overlooked [53]. Furthermore, nesfatin-1 exerts antihyperglycemic effects [54] as it prevents glucose formation and promotes glucose uptake in the liver [55]. Moreover, serum nesfatin-1 plays an important role in regulating β-cell insulin secretion [56] and improves the sensitivity to insulin by up-regulating the insulin receptor and GLUT8 in testis [57]. 

As a result, nesfatin-1 has been proposed as a potential diagnostic biomarker in metabolic disorders, mainly type 2 diabetes mellitus (DMT2). Most studies have underlined that nesfatin-1 levels are significantly lower in DMT2 patients and that there is a negative correlation between nesfatin-1 values and glucose homeostasis parameters, BMI, and atherogenic risk factors [58,59,60]. 

In Table 2, the potential involvement of nesfatin-1 in different pathologies with inflammatory components is summarized, with a special emphasis on its possible clinical relevance.

Figure 1 illustrates the relationship between the tissular expression of nesfatin-1 and the variation in the plasmatic levels of nesfatin-1 in different disorders. 

## 3. Implication of Nesfatin-1 in Digestive Disorders

### 3.1. Gastric Ulcer

Taking into consideration its anti-inflammatory properties, several studies aimed to investigate the potential effects of nesfatin-1 on gastric injury.

Kolgazi M. et al. [90] induced gastric ulcer by subcutaneous administration of indomethacin in rats. Fifteen minutes afterward, some of the rats were treated with nesfatin-1 (intraperitoneally) while the other ones were treated with saline. Serum tumor necrosis factor-α (TNF-α) and interleukin-6 (IL-6) were measured and stomach samples were collected to be microscopically examined and assessed for myeloperoxidase (MPO) activity, malondialdehyde (MDA), glutathione (GSH), and luminol- and lucigenin-enhanced chemiluminescence (CL) levels. The study demonstrated that ulcer induction led to an increase in the serum levels of TNF-α and IL-6 as well as MDA levels and MPO activity, whereas the reduced glutathione (GSH) content had decreased. Moreover, the administration of nesfatin-1 managed to reduce IL-6, CL, MDA levels, and MPO activity, and gastric GSH was restored. Nonetheless, to significantly diminish the TNF-α increase induced by indomethacin, a ten-fold dose of nesfatin-1 was needed. The decreased MPO activity suggests that nesfatin-1 exerts protective activity by inhibiting the recruitment of neutrophils. Nesfatin-1 led to a reduction in the levels of pro-inflammatory cytokines thanks to its antioxidant properties. The decreased levels of TNF-α and IL-1 might be the result of the ability of nesfatin-1 to counteract the up-regulation of mRNA expression of these molecules [91]. The restoration of GSH levels can be explained by the partial reversal of SOD mRNA down-regulation induced by nesfatin-1 [92]. Moreover, the dose administered intraperitoneally in the study managed to delay gastric emptying in comparison to previous research data that suggested that only intracerebroventricular administration had this effect [38]. However, there are no existing data regarding the intragastric administration of nesfatin-1. Furthermore, additional mechanisms have been proposed in other studies. The finding that nesfatin-1 reduces the secretion of gastric acid and increases the mucosal blood flow further supports the gastric protective effects of nesfatin-1 [91]. 

In rat models with gastric ulcers produced by serosal application of acetic acid, nesfatin-1 augmented mucosa regeneration by modulation of the oxidant/antioxidant balance. The inhibition of cyclooxygenase-1 (COX-1) reduced the protective effect of nesfatin-1, while the blockade of cyclooxygenase-2 (COX-2) managed to abolish all the changes induced by nesfatin-1. Hence, its gastroprotective properties involve the regulation of both COX-1 and COX-2 activity with a dominant effect of the COX-2 enzyme, which is of particular importance in ulcer healing [93].

Nesfatin-1 suppressed interleukin-1β (IL-1β) and increased the antioxidant capacity (superoxide dismutase and GSH) with direct effects on neutrophil infiltration and lipid peroxidation. Following these findings, exogenous administration managed to reduce gastric acid secretion and promote mucosal healing in rats with gastric lesions induced by water immersion and restraint stress. The NUCB2/nesfatin-1 levels in plasma as well as the blood flow in the mucosa were increased. At the same time, the study confirmed the functional cooperation between nesfatin-1 and cyclooxygenase-prostaglandin (COX-PG), and it also demonstrated that its potent gastroprotective effects rely on the nitric oxide synthase-inducible nitric oxide synthase (NOS-iNOS) system, vanilloid receptors, and the stimulation of both sensory and vagal nerve fibers. The proposed mechanism is the interaction between calcitonin gene-related peptide (CGRP) and nesfatin-1. In the rats treated with an antagonist of vanilloid receptors, capsazepine, as well as in those with denervation induced with capsaicin, the protective effects of nesfatin-1 were abolished. Therefore, nesfatin-1 probably activates the sensory afferent fibers and increases in this way the release of vasodilatory neuropeptides. CGRP has particularly been proposed to mediate the interaction between nesfatin-1 and vanilloid receptors. In addition, the combined administration of CGRP and nesfatin-1 showed superior protective effects on gastric microcirculation in comparison with the sole administration of nesfatin-1 [91].

### 3.2. Gastric Cancer

In the quest for a novel biomarker for gastric cancer, Wang et.al [94] tried to assess whether nesfatin-1 could potentially be used as a reliable and non-invasive diagnosis tool. Moreover, the presumed correlation between nesfatin-1 and Ki67 protein expression in normal gastric tissue and gastric cancer (GC) tissue was evaluated.

Ki67 is a well-known marker for cell proliferation. Within the cell cycle, it is solely expressed during all the active phases, but it is absent in the resting phase. As a proliferation marker, Ki67 is a close indicator of the proliferation and differentiation of cancer cells, including gastric cancer cells. Moreover, it was recently shown that it might be of great prognostic relevance and that it may consequently be useful to guide treatment strategies [95].

On the other hand, nesfatin-1 was reportedly linked to the mammalian target of rapamycin (mTOR) signaling pathway, which is involved in the dysregulation of cell proliferation. The study included 40 patients with gastric cancer and 40 controls. The results revealed that in patients with GC, the plasma nesfatin-1 levels were significantly elevated. The statistical analysis showed that nesfatin-1 has a 70% sensitivity and 95% specificity to differentiate between patients with GC and healthy individuals, while the most used cancer antigens as part of the diagnosis of GC (carcinoembryonic antigen, carbohydrate antigen 19-9 CA 19-9, carbohydrate antigen CA72-4) have low sensitivity and specificity (approximately 20–30%). Therefore, nesfatin-1 might be of clinical relevance for the screening and diagnosis of GC. Furthermore, the immunohistochemistry analyses showed that the tissue expression of ki67 is up-regulated in GC tissue, and it positively correlated with the plasma nesfatin-1 levels [94].

Gastric cancer is often associated with a higher incidence of depression. As a regulatory neuropeptide and part of the brain–gut axis, nesfatin-1 might be the key element of the relationship between gastric cancer and depressive symptoms. The levels of nesfatin-1 were evaluated both in the plasma and in the brain tissue of mice. The animals were divided into three categories: controls, the gastric cancer group that was not subjected to stress, and the gastric cancer group subjected to stress. In the last group, the levels of nesfatin-1 in tissue and plasma were the highest. The lowest expression was identified in the gastric cancer group not subjected to stress [96]. Taking into consideration the fact that nesfatin-1 is co-expressed with norepinephrine (NE) and 5-hydroxytryptamine (5-HT) in the raphe nucleus of the midbrain [5], it has been suggested that nesfatin-1 stimulates the 5-HT and NE neurons from this area, subsequently activating the corticotropin-releasing hormone (CRF) neurons and the Hypothalamic–Pituitary–Adrenal (HPA) Axis. The HPA is known to be involved in the occurrence of depression, particularly in patients suffering from cancer [97]. 

### 3.3. Necrotizing Enterocolitis

Necrotizing enterocolitis (NEC) is the most common gastrointestinal tract emergency in newborns. Because of its high mortality, new treatments are needed to improve the survival rate of the affected neonates [98]. Considering the anti-inflammatory and anti-apoptotic effects of nesfatin-1, Karadeniz et al. [99] aimed to assess its prospective therapeutic implications on NEC-induced rats.

The newborn rats were induced NEC with a hyperosmolar formula and by exposure to hypoxia. Following the induction, they were administered saline or nesfatin-1 for three consecutive days, and some of the ones treated with nesfatin-1 received capsaicin in order to ablate the afferent nerve fibers. The results indicated that the gene expression of COX-2, nuclear factor kappa-light-chain-enhancer of activated B cells (NF-kB), occludin, and claudin-3 was up-regulated in the NEC rats treated with saline, while the expression level in the nesfatin-1-treated group was not significantly changed. Its implications in the modulation of the inflammatory/anti-inflammatory balance as well as the modifications of the gut microbiome were moderately reversed by capsaicin administration. Of particular interest is the fact that NEC led to dysbiosis by stimulating the growth of Proteobacteria while inhibiting Actinobacteria and Bacteroidetes, and therefore improving the outcome of the affected newborns. The treatment with nesfatin-1 succeeded in counteracting the changes in the composition of the intestinal microbiota.

### 3.4. Mesenteric Ischemia

Intestinal ischemia is a condition characterized by high mortality. The majority of the lesions are caused by the restoration of blood flow resulting in an overabundance of inflammatory cytokines and free radicals [100]. Therefore, the reduction in these inflammatory molecule levels before reperfusion would reduce damage. Tatar et al. tried to investigate both the diagnostic and the therapeutic implications of nesfatin-1 in intestinal ischemia.

Twenty-one rats were divided into three different groups: the first group was subjected to intestinal ischemia for 1 h, followed by a 5-h reperfusion, the second one was exposed to 6-h ischemia, and, in the third group, a laparotomy was performed, without any other additional procedure. Intestine samples were collected for histopathological evaluation as well as intracardiac blood samples to assess a series of biomarkers including nesfatin-1.

The highest nesfatin-1 values were among the second group, while the lowest ones were in the third group. It showed a decrease in endothelial nitric oxide synthase (eNOS) activity and, subsequently, nitric oxide (NO) production. Additionally, there was a positive correlation between the pathology score and the levels of nesfatin-1, suggesting that nesfatin-1 could be an excellent biomarker in acute intestinal ischemia [101]. Similar observations were made by studies conducted on acute mesenteric ischemia-induced rat models, reinforcing the fact that nesfatin-1 reduces oxidative stress index parameters and thus may exert a protective role against ischemia/reperfusion injury [102].

### 3.5. Colitis

Despite its anti-apoptotic and anti-inflammatory properties, the relationship between nesfatin-1 and inflammatory bowel disease has barely been explored. Ozturk et al. were the only ones that approached this topic, having a major focus on the potential underlying mechanisms of action.

Rats were divided into two main groups: colitis and the control group. Under anesthesia, 4% acetic acid was intrarectally administered to induce colitis, whereas the controls received saline solution. Ten minutes afterward, some of the colitis mice received nesfatin-1 intracerebroventricularly. To decipher its mechanisms, three subgroups of the nesfatin-1 group were created 5 min after colitis induction, each of them receiving intracerebroventricularly growth hormone (GH) secretagogue receptor-1a (GHSR-1a) antagonist (antagonist of ghrelin receptors), atosiban (antagonist of oxytocin receptor), and trifluoroacetate salt SHU9119 (antagonist of melanocortin receptors) followed 5 min later by the administration of nesfatin-1 for 3 days. Rats were in the end decapitated and tissue samples were evaluated.

Macroscopic and microscopic damage scores were analyzed histopathologically and they were increased in mice with colitis that did not receive nesfatin-1 treatment. Since atosiban administration abolished both the macroscopic and microscopic healing changes produced by nesfatin-1 administration, nesfatin-1 might act via oxytocin receptors. Moreover, the microscopic damage-reducing properties were significantly diminished following GSHR-1a administration while the macroscopic lesions remained the same. Therefore, ghrelin might modulate the positive effects of nesfatin-1 on microscopic damage. The elevated myeloperoxidase activity, malondialdehyde levels, and luminol and lucigenin chemiluminescence characteristic for inflammation decreased thanks to the administration of nesfatin-1. As a result, nesfatin-1 seems to exert its effects by reducing lipid peroxidation and free radical damage as well as by decreasing neutrophil infiltration. In addition, its beneficial effects were counteracted by atosiban and GHSR-1a, confirming the initial assumption that nesfatin-1 is closely linked to oxytocin and ghrelin receptors. The study outlines a surprising aspect: the association between central nesfatin-1 and peripheral inflammatory status. Thus, colitis and its underlying inflammatory mechanisms might stimulate the activity of CNS nesfatin-1 neurons in order to improve the systemic inflammatory status. Even though the intracerebroventricular administration of nesfatin-1 proved to have positive effects in this regard, further research is needed to understand the link between its central and peripheral activity [103].

A single clinical study conducted by Beyaz S. and Akbal E. [104] has been carried out thus far. It included 17 patients with Crohn’s disease (CD), 18 patients with ulcerative colitis (UC), and 17 healthy patients. The serum levels of nesfatin-1 were detected both in the active and the remission stages along with other inflammation markers including C reactive protein (CRP), erythrocyte sedimentation rate (ESR), and white cell count (WCC). The results showed that nesfatin-1 was notably elevated in patients with CD as well as in patients with UC during the flare-up period. In the remission period, the values moderately decreased but they remained significantly higher in comparison to healthy individuals. Nesfatin-1 showed a high sensitivity (88,2%) and specificity (88,2%) in the active phase of the patients with Chron’s disease. Correspondingly, in patients with ulcerative colitis, the sensitivity was 83.3% and 94.1%. Thus, nesfatin-1 proved to be an excellent novel biomarker that can be used together with other markers as part of the non-invasive diagnosis of IBD. Furthermore, the study tried to assess the association with active phase reactants. 

However, it is still contradictory if nesfatin-1 exerts a pro- or anti-inflammatory role. On the one hand, it has been demonstrated that the neurons expressing nesfatin-1 are stimulated by the presence of a peripheral inflammatory state [105]. Moreover, it was also shown that IL-6 and TNF-α regulate nesfatin-1 production in the human adipose tissue [17]. Thus, the increased levels of nesfatin-1 in IBD can be explained at least partially by the elevated systemic expression of these two inflammatory markers. Despite its obvious implication in several systemic inflammatory diseases, it is still unclear whether nesfatin-1 is part of a pro- or anti-inflammatory response. Taking into consideration its protective role on animal models with colitis and gastric ulcers, one cannot undermine its crucial contribution to the underlying pathophysiological mechanisms.

### 3.6. Pancreatitis

Similar to previous studies on colitis-induced animal models, Buzcu et al. [106] tried to investigate the potential role of nesfatin-1 as an anti-inflammatory marker in acute pancreatitis (AP) as well as the associated fundamental mechanisms. Nesfatin-1 was administered intraperitoneally five minutes before the induction of pancreatitis with two doses of caerulin. Before the pretreatment with nesfatin, atosiban (oxytocin receptor agonist), HS024 (melanocortin receptor agonist), and cortistatin (ghrelin receptor antagonist) were administered. Microscopic damage scores and several biomarkers were determined. MPO, reflecting neutrophil infiltration, malondialdehyde, an end-product of lipid peroxidation, luminol and lucigenin chemiluminescence, serum amylase, lipase, and trypsinogen 2 were increased as part of the AP inflammatory response. However, glutathione levels, showing the antioxidant protective ability, diminished. Nesfatin-1 decreased the oxidative injury, and the way the different receptor agonists/antagonists influenced the biomarkers and the histology scores revealed that nesfatin-1 modulates its activity via melanocortin receptors. 

In addition, another study conducted on rat models with AP sought to evaluate the potential of nesfatin-1 not only as a diagnostic but also as a severity marker. The rats were divided into three groups: the control group, the mild pancreatitis, and the severe pancreatitis group. The serum nesfatin-1, lipase, amylase, and aspartate aminotransferase (AST) and alanine aminotransferase (ALT) levels were measured, and the severity of the induced pancreatitis was evaluated by a pathologist. There was a substantial decrease in nesfatin-1 levels in parallel with the severity of pancreatitis, but no statistically significant correlation was noted. Two main explanations can account for the reduction in nesfatin-1. Nesfatin-1 might be degraded at a higher rate to counteract the high oxidative status in acute pancreatitis. Also, the actual destruction of the pancreatic tissue can result in an evident decrease in nesfatin-1 [107].

Few clinical studies attempted to characterize the connection between nesfatin-1 and AP. In the study performed by Ulger et al. [108], 40 patients with gallstone-induced pancreatitis were enrolled in order to assess plasma nesfatin-1 along with leptin and ghrelin. Two blood samples were collected for each patient, at admission and discharge, respectively. The levels of these hormones were altered in all patients. Nesfatin-1 and leptin were significantly higher at hospitalization in comparison to discharge, whereas ghrelin values had an opposite evolution. Even if the exact mechanisms are still unclear, these findings may be linked with the clinical outcomes in patients with AP.

### 3.7. Cholecystitis

Tekin et al. [109] tried to determine the possible relevance of nesfatin-1 concerning the diagnosis and assessment of acute cholecystitis. The study included four groups: mild, moderate, and severe cholecystitis groups (based on the Tokyo 2018 Guidelines), and the control group. The patients were evaluated for blood nesfatin-1, leukocytes, neutrophils, lymphocytes, and neutrophil/lymphocyte ratio. Nesfatin-1 levels were found to be decreased in the cholecystitis groups compared with the controls, but no statistically significant difference was identified as far as the potential correlation between nesfatin-1 levels and the severity of the disease is concerned. At the same time, the study reinforces the remark that nesfatin-1 production and release are increased in the initial phase to balance out the inflammatory response. As inflammation progresses, nesfatin-1 levels decline. It is noteworthy that nesfatin-1 may vary depending on the severity of the inflammatory response and the type of the affected tissues.

### 3.8. Obstructive Jaundice

The connection between nesfatin-1 and obstructive jaundice (OJ) has barely been addressed thus far. Solmaz et al. [110] studied the effects of nesfatin-1 on OJ in rats. The animals were assigned to three different groups: sham, control, and nesfatin-1. Under general anesthesia, in the sham group, laparotomy and exploration of the common biliary duct (CBD) were performed. The other two groups undertook laparotomy, which was followed by the double ligation of the CBD above the pancreas, which was eventually cut off. Afterward, the groups were treated for 10 days with saline (control group) and nesfatin-1 (nesfatin group). Blood and liver samples were analyzed. 

Biochemical parameters (AST, ALT, alkaline phosphatase, gamma-glutamyl transferase, albumin) were not significantly different within the two groups. As for the cytokine levels, MDA was the lowest and SOD was the highest in the nesfatin group, and the data were of statistical relevance. Thus, nesfatin-1 has a considerable impact on oxidative status. IL-6 levels, TNF-α, and oxidative DNA damage were lower in the nesfatin group yet without being statistically meaningful. However, the histopathological assessment indicated remarkable differences between the controls and the rats treated with nesfatin. The latter showed less neutrophil infiltration of the portal area, hepatocyte necrosis, bile duct proliferation, and edema. Even though most of the inflammation markers determined in the study did not show a critical distinction between the two groups, the histopathological results endorse the beneficial antioxidant and anti-inflammatory potential of nesfatin-1 in OJ. As suggested by the coordinators of the research, a limitation of the study that may have a great impact on the reliability of the results is the relatively short follow-up period. Thus, the relation between the role of nesfatin-1 and the evolution of the disease long-term cannot be foreseen.

## 4. Conclusions

This review underlines the current state of knowledge concerning nesfatin-1, a novel neuropeptide with anti-inflammatory, antioxidant, and antiapoptotic properties. It is expressed in different tissues, both centrally and peripherally, and it plays a crucial role in the pathogenesis of different diseases. As a result, nesfatin-1 can be of great clinical importance as a diagnosis biomarker, severity indicator, and therapeutic agent. However, nesfatin-1 is far from being used as a clinical application in the foreseeable future. The main limitation is the inability to identify its specific receptors. Also, while most findings are encouraging, some results are still contradictory. In particular, the relationship between nesfatin-1 and metabolic diseases is still unclearly defined, with studies leading to opposing results as previously presented in this article review. There might be several reasons behind this, for example, an inadequate selection of the subjects or animal models, faulty assessment methods, a too small sample size, additional unknown factors that can influence the course of the disease studied, inter-individual variability, and the so far not clearly known mechanisms of actions. Hence, nesfatin-1 deserves more attention and further research is clearly needed to explore its diagnostic and therapeutic potential.

## Figures and Tables

**Table 1:** Nesfatin-1: Mechanisms of action.

Pathway	Study Type	Description of the Pathway/Effect	Conclusion	Citation
**AMPK**	Animal models: C57BL/6J mice with induced hepatic steatosis Hepatocyte cultures	Decreased lipogenesis transcription factors PPARγ and SREBP1 In cultured hepatocytes, it stimulates AMPK phosphorylation	Reduces the accumulation of lipids in the liver	Yin et al., 2015 [23]
	Animal model: Male Kunming SPF mice with high-calorie diet + 2 Streptozotocin doses to induce type 2 Diabetes Mellitus	Lowered food intake, insulin resistance coefficient, and blood glucose Nesfatin-1 in low doses stimulates the AMPK-ACC pathway and activates free fatty acid utilization	Inhibits free fatty acid production and stimulates their oxidation	Dong et al., 2013 [24]
	Human colon cancer samples SW480 and SW620 cancer cell lines	In colon cancer tissue samples and SW480 and SW620 cancer cell lines: increased nesfatin-1 expression Nesfatin-1 activated LKB1/AMPK/TORC1/ZEB1 pathway in vivo and in vitro	Nesfatin-1/NUCB2 promotes migration and invasion in colon cancer	Kan et al., 2016 [25]
	Animal model: male Sprague-Dawley rats on normal or a high-fat diet	Activation of AKT/AMPK/TORC2, insulin receptor, and insulin substrate 1 phosphorylation Inhibition of hepatic gluconeogenesis and increased glucose uptake in the muscle	Nesfatin-1 increases insulin peripheral uptake and decreases production of glucose in the liver	Yang et al., 2012 [26]
**ERK/MAPK**	Human gastrointestinal smooth muscle cells (HGSMC)	Up-regulates eNOS activity by involving the ERK/MAPK/mTOR cascade Increases some pro-apoptosis factors (p53 and Fas)	Nesfatin-1 accelerates the apoptosis rate in HGSMC	Tan et al., 2016 [27]
	Rats	Decreases the expression of brain-derived neurotrophic factor (BDNF) and of phosphorylated ERK in the prefrontal cortex and the hippocampus	Nesfatin-1 induces anxiety-like behavior in rats	Ge et al., 2015 [28]
	Male Wistar rats and Zucker fatty rats	Increase in p-ERK1/2 positive neurons in the paraventricular hypothalamic nucleus Increased co-expression in the corticotropin-releasing hormone cells Activation of sympathetic activity in the kidney, liver, and white adipose tissue Hypertensive effects	Nesfatin-1 regulates the autonomic nervous system	Tanida et al., 2015 [29]
	Human SH-SY5Y neuroblastoma cells	Stimulates the protein expression of synapsin-1, p-ERK1/2, and CRH	Nesfatin-1 could be involved in the hypothalamic–pituitary–adrenal axis and in synaptic plasticity	Chen et al., 2018 [30]
**Melanocortin pathway**	Adult male Wistar rats	Enhances the excitability of glucose-sensitive neurons in the lateral parabrachial nucleus Weight loss and increased the expression of uncoupling protein 1 (UCP1) in brown adipose tissue in chronic administration	Nesfatin-1 inhibits food intake and regulates body weight	Yuan et al., 2017 [31]
	Adult male Harlan Sprague-Dawley rats	Nesfatin-1 increases mean arterial pressure	Nesfatin-1 stimulates sympathetic activity	Yosten et al., 2009 [32]
	Ventricular myocytes from adult male Wistar rats	Treatment with nesfatin-1 inhibited L-type Ca^2+^ channels in cardiomyocytes	Nesfatin-1 modulates cardiac performance	Ying et al., 2015 [33]
**AKT**	Wistar rats with isoproterenol-induced myocardial infarction	Nesfatin-1 increases *p*-GSK-3β/GSK-3β and *p*-Akt/Akt expression in the myocardium Nesfatin-1 decreases the levels of troponin-T and proinflammatory cytokines Reduces the apoptotic and necrotic cells	Nesfatin-1 has cardioprotective activity against myocardial infarction	Tasatargil et al., 2017 [34]
	Wistar–Kyoto rats Human aortic vascular smooth muscle cells (VSMCs)	Nesfatin-1 activates PI3K/Akt/mTOR and JAK2/STAT pathways induces VSMCs proliferation and phenotypic transformation Down-regulation of the nesfatin-1 gene inhibited cardiovascular remodeling	Nesfatin-1 is involved in vascular remodeling and hypertension by regulating VSMC proliferation	Lu et al., 2018 [35]
	Sprague-Dawley rats, C57BL/6J mice, and high-fat-diet-induced obese mice Myocytes, hepatocytes, and adipose cells isolated from C57BL/6J mice	Nesfatin-1 increased insulin secretion in vivo and in min6 cells via AKT phosphorylation Nesfatin-1 up-regulated the phosphorylation of AKT in adipose tissue, skeletal muscle, and liver in the mice that received a high-fat diet Nesfatin-1 promotes GLUT4 translocation in the adipose tissue and in the skeletal muscle irrespective of the diet (high-fat or normal diet)	Nesfatin-1 increases the secretion of insulin and the sensitivity to insulin in peripheral tissues	Li et al., 2013 [36]
**CRF pathway**	Adult male Wistar rats	Nesfatin-1 inhibits excitatory neurons involved in gastric distention Nesfatin-1 stimulates the inhibitory gastric distention neurons in the ventromedial hypothalamic nucleus	Nesfatin-1 has implications for digestive disorders and obesity	Feng et al., 2017 [37]
	Adult male Sprague-Dawley rats	Nesfatin-1 decreased dark-phase food intake in case of intracerebral administration, with no effect after ip administration	Nesfatin-1 is involved in the central regulation of food intake	Stengel et al., 2009 [38]
**Ion currents**	Hypothalamic neuronal cultures from Sprague-Dawley rats	Nesfatin-1 increased intracellular Ca^2+^ concentrations in hypothalamic neurons	Nesfatin-1 increases calcium influx in hypothalamic neurons via a G protein-coupled receptor	Brailoiu et al., 2007 [22]
	Beta cells from ICR mice	Nesfatin-1 increases the Ca^2+^ influx in pancreatic beta cells through the L-type Ca^2+^ channels after treatment with glucose	Nesfatin-1 stimulates the release of insulin from beta islet cells in a glucose-dependent manner	Nakata et al., 2011 [39]
	Islet cells from male C57BL/6J mice	Nesfatin-1 inhibits the activity of the Kv channels that stimulate insulin secretion	Nesfatin-1 increases the release of insulin from beta islet cells via the inhibition of Kv channels	Maejima et al., 2017 [40]
	Heart samples from Wistar albino rats	The expression level of the α1c subunit protein of the L-type Ca^2+^ channels in cardiac extracts was elevated in the rats subjected to chronic restraint stress	Nesfatin-1 may be involved in cardiac failure	Ayada et al., 2015 [41]
**NO-cGMP system**	Mesenteric artery isolated from Wistar rats	Nesfatin-1 impairs the production of SNP-induced cGMP, which leads to reduced smooth muscle relaxation	Nesfatin-1 regulates blood pressure by modulating peripheral arterial resistance	Yamawaki et al., 2012 [42]
	Heart samples from Wistar rats	Nesfatin-1 recruits specific GC-receptors (NPR-A—natriuretic peptide receptor A) activating the ERK1/2 and protein kinase G pathways Pretreatment decreases the size of the infarct and lactate dehydrogenase levels	Nesfatin-1 has negative inotropic and lusitropic effects and modulates heart activity Nesfatin-1 protects against ischemia/reperfusion injury	Angelone et al., 2013 [43]

Abbreviations: ACC1—Acetyl-Coenzyme A carboxylase 1; AKT—protein kinase B; AMPK—activated protein kinase; cGMP—cyclic guanosine monophosphate; CRF—corticotropin releasing factor; CRH—corticotropin releasing hormone; eNOS—endothelial NO synthase; ERK—extracellular signal-regulated kinase; GC—guanylate cyclase; GLUT—glucose transporter; GSK-3b—glycogen synthase kinase 3 beta; JAK2—Janus kinase 2; Kv—voltage-gated potassium channels; LKB1—liver kinase B1; MAPK—mitogen-activated protein kinase; mTOR—mechanistic Target of Rapamycin; NO—nitric oxide; p-ERK—phosphorylated extracellular signal-regulated kinase; p-GSK-3b—phosphorylated glycogen synthase kinase 3 beta; PI3K—phosphoinositide 3-kinase; PPARγ—proliferator-activated receptor γ; SNP—sodium nitroprusside; SREBP1—sterol-regulatory element-binding protein-1; STAT—signal transducer and activator of transcription; TORC1—target of rapamycin kinase complex 1; TORC2—target of rapamycin kinase complex 2; ZEB1—Zinc finger E-box binding homeobox 1.

**Table 2:** Implication of nesfatin-1 in multiple disorders.

Group of Disorder	Disease/Process	Type of Study	Observations	Conclusion	First Author and Year
**Neurological** **disorders**	Alzheimer’s disease (AD)	Animal model: *Drosophila melanogaster*	Nesfatin-1 reduces the neurodegenerative process in the eye and bristle of AD-induced models Nesfatin-1 regulates neuromuscular activity Nesfatin-1 reduces the levels of human Tau protein, particularly phospho-tau	Nesfatin-1 impedes neurodegeneration	Yang et al., 2024 [61]
	Parkinson’s disease	C57BL/6 mice	Anti-nesfatin-1 antibody in the lateral ventricle led to: Reduction in the nesfatin-1 level of the cerebrospinal fluid ROS production and depletion of intraneuronal mitochondria Increased membrane permeability, cytochrome c leakage, caspase-3 induced apoptosis	Dopaminergic neuronal depletion is closely correlated with a reduced level of nesfatin-1 in the cerebrospinal fluid	Chen et al., 2021 [62]
	Subarachnoid hemorrhage	Case-control study	Nesfatin-1 levels were increased in the subarachnoid hemorrhage group compared to healthy controls Nesfatin-1 levels positively correlate with the presence, number, and size of the aneurysm	Potential role of nesfatin-1 as a predictive biomarker for acute subarachnoid hemorrhage and the existence of aneurysms	Acik et al., 2020 [63]
**Endocrine and metabolic disorders**	Ischemic stroke	Case-control study	Nesfatin-1 levels were lower in the ischemic stroke group in comparison to controls No significant association between nesfatin-1 levels and the severity of ischemic stroke	Nesfatin-1 may play a role in the pathogenesis of ischemic stroke	Kazimierczak-Kabzińska et al., 2020 [64]
	Feeding Behavior/Obesity	Male Sprague-Dawley rats	Nesfatin-1 decreases food ingestion by reducing the meal size not frequency or the intervals between meals Increase in satiation (size of a meal) under normal weight conditions Increase in satiety (frequency of meals) under diet-induced obesity conditions	Nesfatin-1 has anorexigenic effects	Carr et al., 2015 [65]
		Case-control study	High nesfatin-1 levels in the patients under hemodialysis vs. healthy subjects Significant positive correlation between nesfatin-1 levels and malnutrition inflammation score and the increased interleukin-6 levels Significant negative correlation with body mass index (BMI)	Nesfatin-1 is associated with the nutritional status in end-stage renal disease	Elthakaby et al., 2022 [66]
		Case-control study	Higher serum nesfatin-1 levels in the obese group Nesfatin-1 positively correlates with copeptin, serum insulin level, and homeostasis model assessment of insulin resistance	Nesfatin-1 might be involved in the appearance of insulin resistance Nesfatin-1 regulates food intake	Yin et al., 2020 [67]
		Case-control study	Lower nesfatin-1 level in the metabolic-associated fatty liver disease (MAFLD) group Nesfatin-1 correlated negatively with high-sensitivity-C reactive protein (hs-CRP), alanine aminotransferase, and fasting glucose	Nesfatin-1 could be a new diagnostic biomarker for MAFLD	Afifi et al., 2024 [68]
	Diabetes Mellitus	Case-control study	Nesfatin-1 levels are significantly lower in the healthy obese and diabetic group in comparison to healthy individuals, being the highest in the underweight group	Nesfatin-1 may be part of the common pathogenetic pathway between diabetes and obesity	Samani et al., 2019 [69]
		3T3-L1 mouse adipocytes Human adipocytes (omental visceral adipose tissue)	Nesfatin-1 expression was increased in hypoxic murine adipocytes Nesfatin-1 was highly detectable in the visceral adipose tissue of obese subjects vs. controls Nesfatin-1 levels correlated with binge eating, sweets craving, and hyperphagic behavior	Nesfatin-1 may be an indicator of eating disorders in obese patients	Caroleo et al., 2023 [70]
		Case-control study	Serum nesfatin-1 level was the highest in the patients with diabetes mellitus type 2 (DMT2) Higher nesfatin-1 level DMT2 and pre-diabetic patients vs. controls Nesfatin-1 positively correlated with glucose, insulin resistance, and lipid profile parameters	Nesfatin-1 could be a predictor biomarker for pre-diabetes and DMT2	Matta et al., 2022 [58]
		Case-control study	Higher serum nesfatin-1 levels in gestational diabetes mellitus patients Nesfatin-1 is associated with blood glucose, HOMA-IR. and high-density lipoprotein cholesterol (HDL-C)	Nesfatin-1 has diagnostic value for gestational diabetes	Jiang et al., 2020 [71]
		Case-control study	Lower nesfatin-1 levels in the gestational diabetes mellitus group	Nesfatin-1 is an alternative screening method to the conventional oral glucose tolerance test (OGTT)	Çaltekin et al., 2021 [72]
	Polycystic ovary syndrome (PCOS)	Case-control study	Higher nesfatin-1 levels in PCOS patients Normal nesfatin-1 levels after treatment with Metformin	Nesfatin-1 can be a diagnostic biomarker for PCOS Nesfatin-1 levels can indicate the response to treatment	Mahmood et al., 2023 [73]
		Case-control study	Lower nesfatin-1 levels in PCOS patients Negative correlations between nesfatin-1 levels, visceral adiposity index, BMI, HOMA-IR, and triglyceride levels	Low nesfatin-1 levels may play an important role in the pathogenesis of PCOS	Çaltekin et al., 2021 [74]
**Cardiovascular disorders**	Ischemia/reperfusion injury	C57BL/6 mice H9c2 cardiomyocytes	Ischemia led to down-regulation of NUCB2 transcription with low nesfatin-1 levels Wortmannin (inhibitor of Akt/ERK pathway) abolished the beneficial effects of nesfatin-1 Activation of the Akt/ERK pathway reduces endoplasmic reticulum stress	Nesfatin-1 attenuates ischemia-reperfusion stress	Su et al., 2021 [75]
	Myocardial infarction, angina pectoris	Adult male Wistar rats	Restoration of SOD and the GSH content by nesfatin-1 Nesfatin-1 inhibited autophagy and apoptosis (reduction of caspase 3 and Bax)	Nesfatin-1 could be used to treat myocardial infarction	Naseroleslami et al., 2020 [76]
		Case-control study	Significantly lower nesfatin-1 levels in stable-angina pectoris and acute-myocardial infarction patients No significant difference between the stable-angina pectoris group and the acute-myocardial infarction group Negative correlation between nesfatin-1 low-density lipoproteins, coronary atherosclerosis status (Gensini score), troponin T, and creatine kinase-MB	Nesfatin-1 is involved in the pathogenesis of atherosclerosis and myocardial infarction	Nejati et al., 2021 [77]
	Coronary artery disease (CAD)	Case-control study	Low nesfatin-1 levels in the CAD groups (stable chronic CAD and unstable angina) vs. controls Nesfatin-1 correlated negatively with the Gensini score used to quantify the severity of CAD Nesfatin-1 is closely associated with high-sensitivity-C reactive protein (hsCRP) levels	Nesfatin-1 is negatively associated with the presence and severity of CAD	Kadoglou et al., 2021 [78]
	Normotension, Hypotension	Male Sprague-Dawley rats	Nesfatin-1 increases catecholamine, vasopressin, and renin concentrations Significant increase in the mean arterial pressure in both normotensive and hypotensive rats	Nesfatin-1 has pressor effects and regulates sympathetic activity	Yilmaz et al., 2015 [79]
**Rheumatologic disorders**	Rheumatoid arthritis (RA)	Case-control study	Positive relationship between nesfatin-1, IL-1β, and TNF-alfa levels in synovium and synovial fluid samples Nesfatin-1 synovial levels correlated positively with rheumatoid factor Nesfatin-1 has a sensibility of 77.5% and a specificity of 60% in differentiating patients with RA from healthy individuals	Synovial nesfatin-1 is involved in the inflammatory process in RA and correlates with the severity of the disease	Zhang et al., 2021 [80]
		Case-control study	Nesfatin-1 serum level was higher in the RA group High nesfatin-1 level correlates with CRP and N-terminal propeptide of type I procollagen (P1NP), a biomarker of the formation of bone matrix	Nesfatin-1 might regulate the function of osteoblasts in RA patients	Seewordova et al., 2022 [81]
		Synovial fibroblasts from patients with RA	Higher expression of NUCB2 mRNA in the synovium in the patients with RA The high expression of NUCB2 correlated with other genes controlling DNA replication, intercellular adhesion, and the interaction with the surrounding extracellular matrix	Nesfatin-1 is involved in the pathogenesis and progression of RA	Zhang et al., 2022 [82]
**Reproductive disorders**	Testicular function	Mice testis	Nesfatin-1 had an increased stimulatory role in testosterone production Nesfatin-1 increased intratesticular glucose transport It accelerated cell proliferation and decreased apoptosis; higher sperm production	Nesfatin-1 regulates testicular function	Ranjan et al., 2019 [83]
	Puberty	Male parks strain mice Testes from pre-pubertal mice	Nesfatin-1 facilitated GnRH-R expression, the production of steroid hormones, spermatogenic biomarkers (Bcl2, PCNA), glucose-metabolism-related proteins (GLUT8, insulin receptor)	Nesfatin-1 facilitates spermatogenesis and steroidogenesis Nesfatin-1 promotes pubertal transition	Ranjan et al., 2019 [84]
	Testicular torsion	Male Sprague-Dawley rats	Nesfatin-1 induced a lower percentage of spermatozoa with head defects and lower levels of pro-inflammatory cytokines: TNF-α, IL-6, caspase-3	Nesfatin-1 prevents the degeneration of spermatogenic cells induced by torsion	Arabaci et al., 2018 [85]
	Pregnancy/Abortion	CBA⁄j female mice, BALB⁄c male mice, and DBA⁄2 male mice	The level of nesfatin-1 was higher in the uteri of the abortion animals The expression in the uterus might be down-regulated by cytokines and chemokines in pregnancy	Nesfatin-1 expression might be involved in the maintenance of pregnancy	Chung et al., 2017 [86]
**Other diseases**	Acute lung injury (ALI)	Mice BEAS-2B human alveolar epithelial cell line	Administration of nesfatin-1 reduced oxidative stress and inflammation both in mouse lung tissue and BEAS-2B cells Nesfatin-1 inhibits inflammatory pathways downstream of HMGB-1	Nesfatin-1 ameliorates inflammation in ALI	Wang et al., 2020 [87]
	Osteoarthritis (OA)	Sprague-Dawley rats	Nesfatin-1 inhibits MAPK, the IL-1β activation of NF-kB and Bax/Bcl-2 signaling pathways in chondrocytes Nesfatin-1 has anti-inflammatory functions and reduces the levels of MMPs, caspase-3, COX-2, and IL-6	Nesfatin-1 has a protective role in OA	Jiang et al., 2020 [88]
	Depression	Case-control study	Nesfatin-1 serum levels significantly higher in adolescents with major depressive disorders Positive correlation between nesfatin-1 levels and Hamilton Depression Rating Scale (HAMD-17) used to assess the severity of the disorder	Nesfatin-1 may be a diagnostic and disease-severity biomarker for depression	Sun et al., 2023 [89]

Abbreviations: AKT—protein kinase B; Bax—Bcl-2-associated X Factor; Bcl-2—B-cell lymphoma 2; BMI—body mass index; CRP—C reactive protein; DAS28—disease activity score for 28 joints; ERK—extracellular signal-regulated kinase; ESR—erythrocyte sedimentation rate; FSH—follicle-stimulating hormone; GnRH-R-Gonadotropin-releasing hormone receptor; GSH—reduced glutathione; HOMA-IR—Homeostatic Model Assessment for Insulin Resistance; HMGB1—high mobility group box 1; IL-6—interleukin-6; Interleukin-1β—IL-1β; LH—luteinizing hormone; iNOS—inducible nitric oxide synthase; MAPK—mitogen-activated protein kinase; MMPs—of matrix metalloproteinases; MPP—1-Methyl-4-phenylpyridinium ion; MPTP—1-Methyl-4-phenyl-1,2,3,6-tetrahydropyridine; NF-kB—Nuclear factor kappa-light-chain-enhancer of activated B cells; PCNA—Proliferating cell nuclear antigen; PLA2—phospholipase A2; RAF—rapidly accelerated fibrosarcoma; ROS—reactive oxygen species; SOD—superoxide dismutase; TNF-α—tumor necrosis factor-α.

## Data Availability

No new data were created or analyzed in this study. Data sharing is not applicable to this article.
